# Animal health and nutrition: metabolic disorders in cattle and improvement strategies

**DOI:** 10.3389/fvets.2025.1470391

**Published:** 2025-03-25

**Authors:** Danju Kang, Shera Elizabeth Lungu, Felix Danso, Chrystella Fernanda Dzou, Yanjun Chen, Xinyu Zheng, Fanghong Nie, Hongying Lin, Jinjun Chen, Guangxian Zhou

**Affiliations:** ^1^Department of Veterinary Medicine, College of Coastal Agriculture, Guangdong Ocean University, Zhanjiang, Guangdong, China; ^2^Department of Economics, College of Applied Economics, Guangdong Ocean University, Zhanjiang, Guangdong, China; ^3^College of Food Science and Technology, College of Coastal Agriculture, Guangdong Ocean University, Zhanjiang, Guangdong, China; ^4^Department of Animal Science, College of Coastal Agriculture, Guangdong Ocean University, Zhanjiang, Guangdong, China

**Keywords:** metabolic disorders, ketosis, milk fever, negative energy balance, hepatic lipidosis

## Abstract

The health and productivity of cattle are significantly compromised by metabolic diseases on a global scale. These disorders disrupt normal metabolic processes, leading to substantial economic losses for the livestock industry. Metabolic disorders can arise from defective biochemical pathways, deficiencies in enzymes, coenzymes, or cofactors, and may be either inherited or acquired. Dairy cows are particularly susceptible during the transition period from late lactation to early management, facing conditions such as ketosis, milk fever, and hepatic lipidosis. This susceptibility is primarily due to reduced dry matter intake caused by fetal development and a decline in rumen capacity. The negative energy balance (NEB) during this phase, characterized by elevated blood concentrations of non-esterified fatty acids (NEFAs) due to increased energy mobilization, is closely linked to the onset of these diseases. Providing high-energy-density diets during this period is critical to mitigating the effects of NEB. Metabolic disorders represent a major health challenge in cattle, adversely affecting animal welfare and agricultural output. A comprehensive understanding of their etiology, clinical manifestations, diagnostic approaches, and management strategies is essential for effective prevention and treatment. Ongoing research and the adoption of preventive measures are vital to reducing the economic and health impacts of these diseases. Early diagnosis and proactive management strategies are crucial to mitigating their impact on dairy cattle health and productivity. Early identification enables timely interventions, preventing disease progression and reducing adverse effects on animal health. Proactive measures, such as optimizing nutritional programs, implementing precision farming technologies, and ensuring timely veterinary care, are essential for enhancing the overall wellbeing of dairy cows. This review serves as a valuable resource for veterinarians, researchers, and dairy farmers, offering in-depth insights into the etiology, clinical signs, diagnostics, and management of prevalent metabolic disorders in dairy cattle. By equipping stakeholders with this knowledge, it aims to support informed decision-making and improve herd management practices. The focus on early diagnosis and proactive strategies underscores the potential to significantly reduce the economic and health burdens imposed by metabolic diseases on the livestock industry.

## 1 Introduction

This article provides a brief overview of the most significant metabolic diseases affecting dairy cows during this crucial transitional phase. This review discusses key metabolic diseases such as hypomagnesemia, milk fever, ketosis, and fat cow syndrome. Even on well-managed farms, these illnesses are becoming a greater concern for the dairy sector because they often cause both overt and subtle financial losses for farmers (1, 2). This is due to the fact that they have a direct effect on the quantity and quality of milk produced the rate at which the animals produce, and the overall profitability of dairy farming. Metabolism is the sum of physical, chemical, and metabolic processes involved in the absorption, breakdown and synthesis of essential organic molecules within the body. During metabolic activities, numerous metabolites are released, which are either used as building blocks or broken down and eliminated as waste. Nutrients are converted into energy that the body's cells, organs, systems and the entire organism utilize for regular functions (3). This metabolism encompasses all activities necessary for an organism to survive and operate normally. A disruption in any metabolic process can lead to malfunctions in different parts of the body or the entire organism. Hence, metabolic diseases or disorders are defined as disruptions of one or more metabolic processes related to the control of certain metabolites in bodily fluids (3). Livestock disorders, known as metabolic diseases, are caused by productivity methods and arise when the body's energy, calcium or magnesium stores are insufficient to fulfill the demands of metabolism (4).

A number of physiological changes occur during the transition phase, including the rapid growth of the fetus, decreased volume of the rumen, development of the mammary gland for the synthesis of milk after calving, social changes, and modifications to the cow's living environment (5). In conclusion, the amount of dry matter consumed by cows can be reduced by up to 40% during the transition phase, and their nutritional requirements may increase significantly by up to three times for glucose and twice for amino acids (6). Lack of vitamins A and E causes NEB (7). Possible explanations for some of the findings of Buonaiuto et al. (8), which may be related to negative NEB status observed during the periparturient phase. According to Plaizier et al. (9), a negative nitrogen balance can also occur in cows in the initial days following calving, in addition to NEB. The energy imbalance in this phase cannot be met by dairy cows that consume more feed (10). High plasma levels of the anorectic hormone leptin, which is directly linked to a high loss of body condition caused by intense lactogenesis, are characteristic of lactating dairy cows, according to Straczek et al. (11). Therefore, cows are forced to use up bodily reserves, such as muscle and fat. During the peripartum period, mammals are vulnerable to several metabolic disorders and physiological instabilities, which may hinder their productivity (12–14). Dairy cows experience increased inflammation and a malfunctioning immune system during the peripartum period because of changes in immunological, metabolic, and endocrine system pathways (15).

The amount of energy used roughly doubles within a few days. Cattle experience a period of negative energy balance when their energy intake is insufficient to meet their needs. These metabolically stressed times are linked to several illnesses or conditions collectively referred to as metabolic disorders. These are rather prevalent; roughly during calving, 30%−50% of dairy cattle are thought to be afflicted by an infectious or metabolic condition (16).

Cattle in the early stages of lactation experience a negative energy balance, or NEB, as a result of a sharp rise in nutrient requirements to sustain milk production that outpaces increases in food intake. The mobilization of stored body energy supplies extra energy. Concurrently, there are decreased calcium and phosphorus levels, as well as elevated blood concentrations of β-Hydroxybutyric Acid (BHBA) and NEFAs, as noted by Drackley (17). Asserted that the process of uncontrollable mobilization of lipids in response to high NEB during early lactation is responsible for several health issues in dairy cattle. The risk of ketosis, hepatic lipidosis, hypocalcemia, and infectious illnesses, including metritis and mastitis, is increased by these metabolic alterations (18–20).

**Figure 1:**
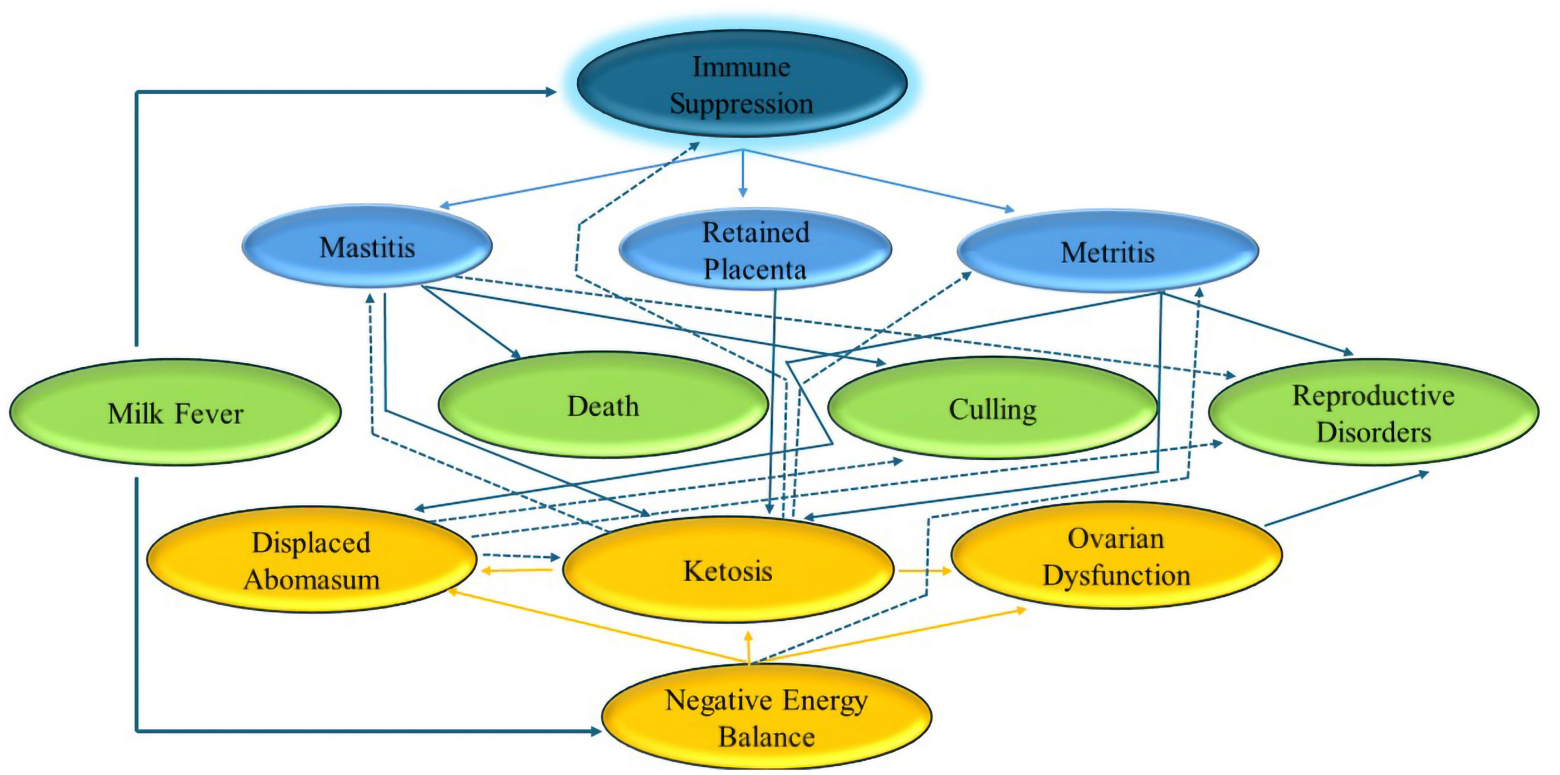
Summary of interplay of metabolic disorders. Based on Overton (127).

Increased levels of NEFAs in blood are associated with lower feed intake. NEFAs may directly (21) or indirectly (22) decrease neutrophil function. Cattle frequently undergo significant oxidative stress during the early stages of lactation because of their high metabolic demands and pathogen threats (23, 24). This condition also exacerbates the pro-inflammatory state which compromises immune function. These conditions not only occur around calving during the same risk period but are also linked together (25). Cattle are more likely to develop further metabolic disorders if they already have at least one metabolic disorder. Figure 1 shows some of these connections.

Metabolic disorders are also common in dairy herds. Despite producing milk, 45%−60% of dairy cattle experience metabolic disease development during the transition phase, which starts during the peripartum period and lasts for many weeks throughout the early weeks of lactation. During this time, several changes occur that present a metabolic challenge for cows: hormonal changes, transitioning the animal from a non-lactating to a lactating state, a significant reduction in feed intake, and a change in diet from a roughage-based diet (grass and hay) to one that is heavy in quickly fermentable carbohydrates (high-grain diets).

Cow fertility, longevity, general health, and lactate production ability can all be significantly affected by metabolic abnormalities (26). During the transition from the dry period to lactation, most metabolic issues occur during calving. Because genetics is increasing milk production, metabolic problems remain a significant risk, despite ongoing improvements in the diet and transition management of cows. Ketosis, particularly subclinical ketosis resulting from NEB, is the most prevalent metabolic condition that has the greatest effect on production transition cows. A costly condition, subclinical ketosis affects the immunological system, reproductive capacity, and milk output of dairy cows over the course of their lactation. According to data, subclinical ketosis might result in a 20% reduction in dry matter intake (DMI) and an average 2 kg drop in milk output. All of these diseases result in financial losses due to lower milk output, decreased milk production efficiency, early culling, veterinary expenses, decreased fertility, and in severe cases, death. Ketosis is one of the most expensive metabolic conditions to treat.

## 2 Fat cow syndrome

A typical metabolic issue in dairy cow production during the transition from the dry period to lactation is fat cow syndrome, also known as fatty liver disease. This syndrome develops when NEFAs are absorbed by the liver and accumulate as excess triacylglycerols (TAGs). The buildup of fat, primarily triacylglycerols, in hepatocytes is known as fatty liver. Long-chain fatty acids are first released from adipose tissue and subsequently taken up by the liver to create them. Liver steatosis is categorized as mild, moderate, or severe based on the amount of fat in the liver (27). An insufficient amount of energy causes fat metabolism to increase, resulting in the accumulation of extra fat in the liver. This condition is the main metabolic illness that affects dairy cows during the first stages of lactation. TAGs are the form in which fats generated from adipose tissue are stored in liver cells after being transported there by the blood. An abrupt spike in energy requirements during the first stages of lactation is the cause of this disease. It can suppress the appetite of obese pregnant animals, particularly twin pregnancies, in beef cows (28). According to Andrews et al. (29), it may result in infertility, down cow syndrome, retained placenta, metritis, mastitis, and ketosis. Alterations in blood enzyme activity can arise from increased cellular activity and cell injury. Early lactation significantly increases serum aspartate transaminase (AST) activity compared to late pregnancy (*P* < 0.05), indicating hepatocyte injury from steatosis and release of the enzyme into the blood. Liver damage caused by fat infiltration is associated with liver enzyme activity in blood. AST is the most accurate measure of liver health during the transition. It has been shown that early lactation causes an increase in AST activity. This disorder affects obese periparturient cows and causes a combination of digestive, reproductive, metabolic, and infectious problems.

### 2.1 Etiology

**Figure 2:**
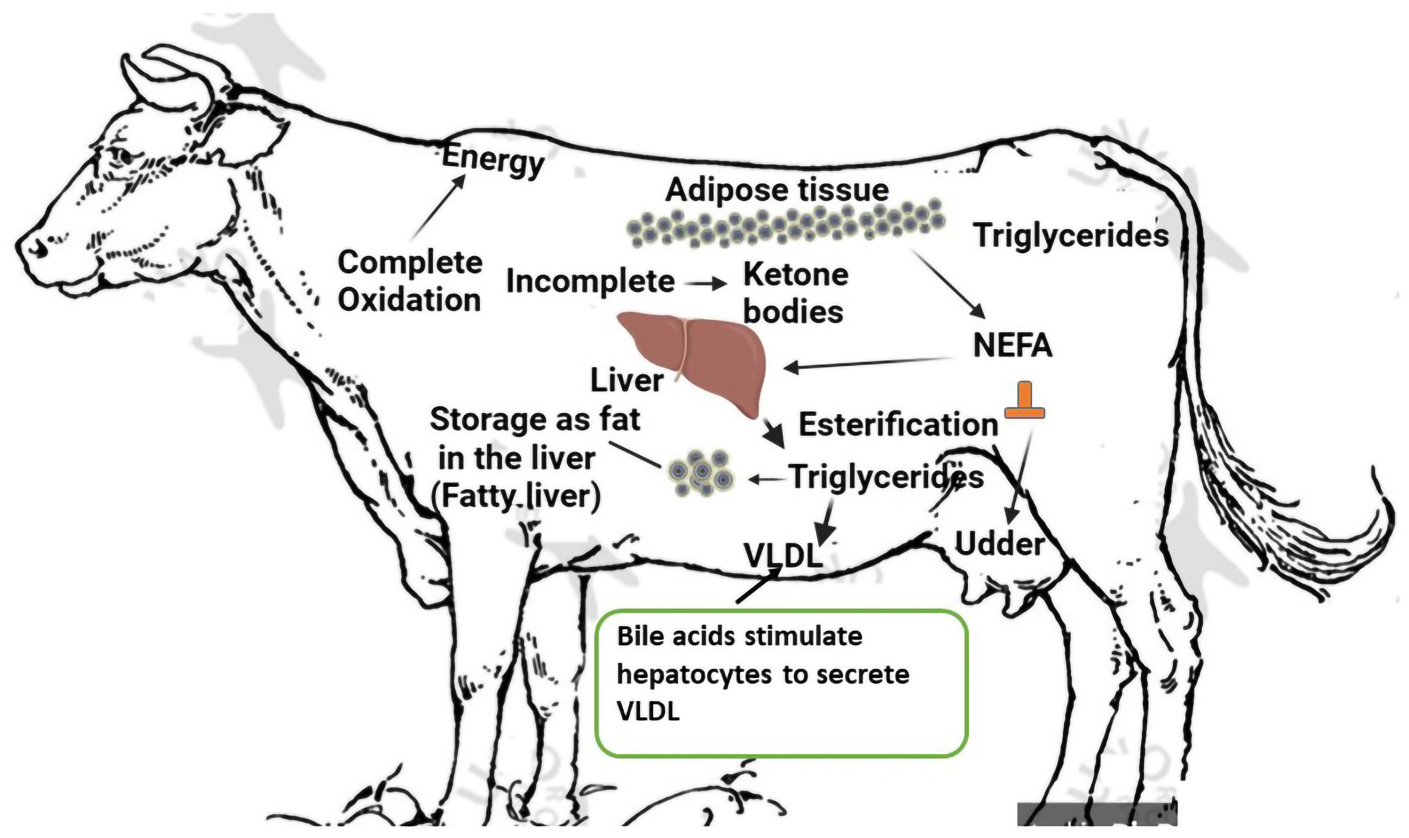
Etiology of fat cow syndrome. Created with the web-based BioRender tool (BioRender.com).

When NEFAs from adipose tissue are mobilized beyond the liver's capacity for lipid oxidation and secretion, the result is the accumulation of TAG in the liver (30). Due to the increased sensitivity of adipose tissue to lipolytic chemicals and decreased sensitivity to lipogenic compounds, cows with fatty livers have larger adipose stores and mobilize more TAG, which raises plasma NEFAs concentrations. Additionally, as demonstrated by decreased plasma apolipoprotein and lipid concentrations and decreased serum lecithin-cholesterol acyltransferase (LCAT) activity, cows with fatty livers have decreased fatty acid oxidation, hepatic apolipoprotein synthesis, and lipid secretion (31). Cows with fatty livers also have challenges with glucose metabolism in addition to problems with lipid metabolism. According to De Koster and Opsomer (32), cows with fatty livers are either hypoinsulinemic hypoglycemic or hyperinsulinemic-hyperglycemic because of decreased hepatic gluconeogenesis or decreased insulin and glucagon secretion, which indicates insulin resistance. Furthermore, there is a drop in plasma amino acids. In conclusion, cows with fatty livers have less glucose, amino acids, and lipids available for peripheral tissues. Fatty liver can form within 24 h after an animal stops eating. This usually occurs around the time of calving. Until the cow achieves a positive energy balance, which may take more than 10 weeks after calving, especially if the fatty liver is severe, the amount of fat in the liver does not decrease once it has been deposited. The risk of fatty liver disease is significantly higher in overweight cows (Body Condition Score > 3.5). In the case of energy deficiency, stored fat is released in the form of free fatty acids, which can be used as a source of energy or oxidized to triglycerides in the liver, where they are accumulated or transported as very-low-density lipoproteins (VLDL). Due to the limited possibilities of synthesis of triglycerides and their transport as VLDL, a large amount of released fat results in fatty liver as fat accumulates in liver cells. Serum fatty acid levels increase from about 2 weeks before calving, peaking up to 2 days after calving, and return to normal levels in the 3^rd^ week of lactation (33). A fatty liver is associated with a negative energy balance, which is normal during the first few weeks after calving. Fatty liver occurs when metabolism cannot be adjusted to meet the requirements of the body. Under normal physiological conditions, the level of fat in the liver rises a few weeks before calving to approximately 20% in the 1^st^ week after calving, and slowly decreases to < 5% by week 26 post-partum. However, the difference can range from almost 0% to 70% in the 1^st^ week after calving. Fat mobilization begins 2–3 weeks before calving and is most likely initiated by hormonal changes caused by parturition, not by energy deficiency. Liver changes are functional, reversible, and dependent on metabolic requirements during late pregnancy and early lactation. In cows with experimentally induced fatty livers, the intensity of liver glycogenesis during the perinatal period is higher than that in cows without steatosis. Low glucagon content leads to low blood glucose concentrations, low insulin levels, and high fatty acid mobilization, causing severe fatty liver disease. Figure 2 illustrates the etiology of fat cow syndrome.

#### 2.1.1 The relationship between fat reserves and the ovarian cycle

In dairy cattle, the anestrus phase after parturition is prolonged in proportion to an increase in adipose mobilization. Furthermore, compared to cattle in good physical condition, extremely underweight cows typically experience delayed initial estrus following calving (28). Owing to disrupted internal hormonal signaling, cows with fatty livers are unable to maintain pregnancy, which lowers fertility. After calving, estrogen levels are hundreds of times greater than during estrus, and this can lead to an increase in TAG synthesis in the liver (27).

### 2.2 Clinical presentation

The syndrome has no unique clinical signs; however, a distinctive indication is an overly fat cow. Other disease characteristics include the presence of inappetence, depression, and low milk production, excessive loss of body condition, progressive debilitation, and general body weakness. However, there is a severe incidence of milk fever, ketosis, clinical mastitis, and retained fetal membranes. Blood test results that show elevated levels of NEFAs concentrations and increased ketones.

### 2.3 Clinical diagnosis

Blood and liver tissue samples obtained from cows following abdominal surgery, during a skin biopsy of the liver, or in the abattoir immediately after slaughter have been used to characterize the biochemical alterations linked to fatty liver disease. Depending on the fatty liver, different biochemical indicators are present. With a higher fat content in the liver, it's activity increases (27). Blood tests, particularly for liver enzyme levels, are necessary to evaluate the state of the liver. According to Gerspach and Ruetten (34), the activity of these enzymes increases when steatosis damages the liver cells. Decreases in blood glucose, total protein, albumin, globulin, cholesterol, triglycerides, and urea are caused by decreased enzyme production in the liver with a substantial fatty liver. Furthermore, the secretory capacity of the hepatocytes is diminished, which increases blood levels of bile acids, ammonia, and total bilirubin (35). According to Andrews et al. (29), there is a drop in leukocyte levels, glucose, cholesterol, albumin, magnesium, insulin, and free fatty acids and an increase in bilirubin and AST. In a study involving 59 cows, gamma-glutamyltransferase (GGT), (AST), succinate dehydrogenase (SDH), and total bilirubin levels were at least twice the normal range, indicating fatty liver in 50% of the cows and the need for treatment for secondary ketosis in 50% of them. To determine the fatty liver and triglyceride contents, a liver biopsy can be performed. This is the most accurate way to define this illness. In healthy cows, the percentage of triacylglycerols in the liver ranges between 10% and 15%. Biochemical or histological techniques can be used to determine the lipid content of liver tissue by biopsy. Both offer trustworthy results for liver fat content (28). The following categories are distinguished: normal liver or mild steatosis (0%−20% lipids), moderate fatty liver (20%−40%), and severe fatty liver (>40%) based on the level of degeneration and fat infiltration in the hepatocytes. Owing to damage to the cell membrane, fatty liver causes hepatocytes to degenerate, releasing cytoplasmic enzymes (AST, GGT, and LDH) and increasing their total activity in the bloodstream (35).

### 2.4 Treatment

There is practically no satisfactory outcome when treating cows with clinically severe fatty liver syndrome. Several treatment plans have been suggested, as is the case for the majority of illnesses for which there is no proven cure. Treatment during a crisis, treating the diseased liver, and preventing further occurrences will be the three areas of therapeutic approach that will be discussed.

#### 2.4.1 Treatment during a crisis

While confirming the diagnosis of fatty liver, treat the cow as usual for mastitis, milk fever, or any secondary issues. The recommended duration of therapy is seven to 10 days or longer, and should be somewhat intensive. Large doses of 10% or 50% dextrose IV administered preferably slowly via a drip at a rate of 60.0 g/h, or the equivalent dosage twice or thrice a day, are necessary for the therapeutic effect of glucose. Potassium chloride and insulin can be administered to combat cellular glucose intolerance (36). Every 36.0–48.0 h, 150–200 units of protamine zinc insulin are administered subcutaneously. It is recommended to use adrenocorticotropic hormone rather than exogenous corticosteroids to promote endogenous corticosteroid secretion. Intramuscular ACTH doses are administered as follows: 400.0 units on days two and three, no units on day four, and 200.0 units on day five. Along with ACTH therapy, 1–2 quarts of propylene glycol are administered orally each day as a prelude to glucose. Although their effectiveness in treating fatty liver crisis has not been established, a number of vitamins, liposome, amino acids, minerals, appetite stimulants, and other nutrients and non-nutrient substances have been tried and suggested. To promote the clearance of fat from the liver, a dose of 50.0–100.0 g of choline chloride per day for 3 days has been suggested. Therefore, selenium and vitamin E might be useful antioxidants (37). Oral cobalt sulfate solution and/or B- complex vitamins are frequently used to stimulate appetite. Given that cows with fatty liver are more prone to infectious diseases, antimicrobial medications are recommended to help avoid subsequent infections during the healing process. However, choosing antimicrobial drugs that are less reliant on liver activation or elimination may be beneficial.

#### 2.4.2 Treating the diseased liver

Bovine fatty liver disease has various potential therapies, as is the case with many diseases for which pharmacological therapy is ineffective. The capacity of the liver to return to normal function is significantly compromised. The veterinarian should use caution while administering medication to cows with suspected or proven liver disease (38). The management of fatty liver syndrome appears to benefit from ACTH (38) as been demonstrated experimentally that fatty liver disease is caused by a deficiency of highly unsaturated phospholipids. The cofactor necessary for the synthesis of these vital phospholipids is choline (39). To remove fat from liver cells, slow down the deposition of fat, or stop further damage, lipotropic drugs such as choline and methionine may be utilized. For adult dairy cows, a recommended dosage of 1.0–8.0 g of choline and 20.0–30.0 g of methionine is given. With the exception of choline, which is unquestionably important in the therapeutic care of fatty liver syndrome, there is little evidence to support the use of lipotropic drugs when there is no established deficit of the agent in question (40, 41).

Although endogenous or exogenous corticosteroids aid in the reduction of fat deposits, enhance gluconeogenesis, and are advantageous for fatty liver cows, they also tend to counteract the effects of insulin, lower the rate at which cells use glucose, and accelerate the breakdown of proteins (42). Milk production and plasma corticosteroid levels have been demonstrated to be inversely correlated; however, it is unclear how artificially elevated endogenous or exogenous corticosteroid levels affect milk production. When it comes to the treatment of animals with liver illness, vitamin K is essential. Vitamin K is stored in the normal liver and can be severely reduced in liver disease. B-complex vitamins are occasionally administered to animals with fatty liver disease to boost their appetite and supply cofactors for metabolism. Selenium and vitamin E are occasionally utilized because of their antioxidant properties, which aid in protecting the liver. A low-protein, high-energy diet is crucial for the management of fatty liver diseases. Therefore, force feeding animals might be necessary. In the event that the affected cow lives, it ought to have access to pasture or hay for recovery. If the sickness causes depletion of the body's potassium levels, oral potassium supplementation may be necessary.

### 2.5 Prevention

For disease prevention, potential risk factors of the disease must be reduced or eliminated. Early diagnosis and treatment that affects voluntary feed intake in late pregnancy and shortly after calving are essential for reducing fat mobilization to cover the energy requirements during periods of negative energy balance and to maintain or increase liver glycogenesis. To prevent fatty liver, it is imperative to promptly address conditions such as ketosis, abomasum displacement, retained placenta, mastitis, hypocalcemia, and down cow syndrome. A restricted diet throughout the dry season is the primary component of prevention to guarantee a suitable body condition for calving, that is, BCS of 2.5–3.0 Preventing the breakdown of fat and fatty liver would involve ensuring that cows are calving at the appropriate body condition. A calf's optimal body condition score is in the range of 2.5 to 3.0. At this point, cows should be dried off and their weight should be maintained during the dry season. Reducing stress is crucial to avoid fatty liver disease. Avoiding abrupt changes in surroundings is recommended. For instance, alterations to diet, housing, temperature, and herd mates may result in a decrease in the amount of feed consumed and initiate catecholamine-mediated increases in the mobilization of fat.

## 3 Ketosis

Ketosis is a prevalent metabolic disease in dairy cows that often occurs 2–4 weeks after calving. It is distinguished by an elevated level of BHBA in the blood, milk, and urine (26, 43). Cows that have clinical ketosis display signs such as anorexia, abnormal licking and chewing, rapid weight loss, and decreased milk output (44). Ketones are metabolic byproducts resulting from the conversion of lipids into carbohydrates. Ruminants utilize ketones as an energy source to a restricted degree within normal physiological conditions (45). It is essential to decrease the severity and duration of NEB in order to prevent ketosis (46). Cows may suffer from subclinical ketosis (SCK) and clinical ketosis (CK), with the main difference lying in the manifestation and severity. SCK is a subclinical state where cows show no obvious clinical symptoms, but the level of ketone bodies, such as BHBA, is elevated (typically BHBA > 1.2 mmol/L), usually detected through laboratory tests or on-farm monitoring tools. In contrast, CK is a more severe condition characterized by obvious symptoms such as decreased appetite, weight loss, reduced milk production, and even neurological signs. Recent research has focused on understanding the mechanisms, diagnosis, and prevention of these conditions. These advancements aim to improve early detection, prevention, and overall herd health, thereby enhancing dairy cow productivity and welfare. To better understand the differences between clinical and subclinical ketosis, including their diagnosis, treatment, and prevention, Table 1 provides a comprehensive comparison.

**Table 1:** Diagnosis and treatment of clinical ketosis and subclinical ketosis in dairy cows.

**Aspect**	**Clinical ketosis**	**Subclinical ketosis**
Definition	A significant metabolic disorder with visible clinical signs	No obvious clinical signs, but elevated ketone bodies in the blood
Incidence rate	Lower (approximately 2%−10%)	Higher (approximately 10%−40%)
Clinical signs	- Reduced or loss of appetite - Decreased milk production - Weight loss - Depression or neurological signs (e.g., circling, licking objects) - Acetone odor in breath or milk	No obvious clinical signs, but may have slight declines in production performance
Diagnostic methods	- Observation of clinical signs - Blood BHBA test (>3.0 mmol/L) - Urine or milk ketone test (strong positive)	- Blood BHBA test (1.2–3.0 mmol/L) - Milk or urine ketone test (weak positive)
Treatment	- Intravenous glucose infusion (500.0 ml of 50% glucose solution) - Oral propylene glycol (300.0–500.0 ml/day for 3–5 days) - Supplementation with vitamin B12 and corticosteroids (if necessary) - Adjust diet to increase digestible carbohydrates	- Oral propylene glycol (200.0-300.0 ml/day for 3–5 days) - Adjust diet to optimize energy balance - Supplementation with vitamins and minerals
Prevention	- Formulate a balanced diet to avoid negative energy balance - Regularly monitor blood or milk ketone levels - Improve management during the transition period to reduce stress	- Optimize nutrition management during the transition period - Regularly monitor ketone levels - Provide high-quality forage and appropriate concentrates
Prognosis	Good prognosis with timely treatment, but may affect production and reproductive performance	Good prognosis with early intervention, effectively preventing progression to clinical ketosis

### 3.1 Etiology

Clinical ketosis and subclinical ketosis are metabolic disorders primarily caused by NEB in dairy cows during early lactation. This occurs when the energy demands for milk production surpass the energy intake from the diet, leading to the mobilization of body fat reserves. The liver metabolizes these fats into ketone bodies, such as BHBA, acetoacetate, and acetone, which accumulate in the blood, urine, and milk. Subclinical ketosis is characterized by elevated ketone levels (BHBA 1.2–3.0 mmol/L) without overt clinical signs, while clinical ketosis presents with visible symptoms such as reduced milk yield, weight loss, and neurological abnormalities, alongside higher ketone concentrations (BHBA > 3.0 mmol/L). Subclinical ketosis in cows involves ketone body accumulation with no obvious symptoms. Blood ketone levels rise, the liver faces metabolic stress as fatty acid processing is disrupted, and there are subtle performance impacts. In clinical ketosis, hypoglycemia, severe liver impairment, and increased depression occur, with body defenses against negative energy balance severely compromised. Key risk factors include poor dry period management, over conditioning, inadequate transition diets, and stressors such as overcrowding or concurrent diseases like mastitis or metritis. Genetic predisposition may also influence susceptibility. Effective prevention focuses on optimizing nutrition, monitoring ketone levels, and ensuring proper herd management during the transition period (47, 48).

**Figure 3:**
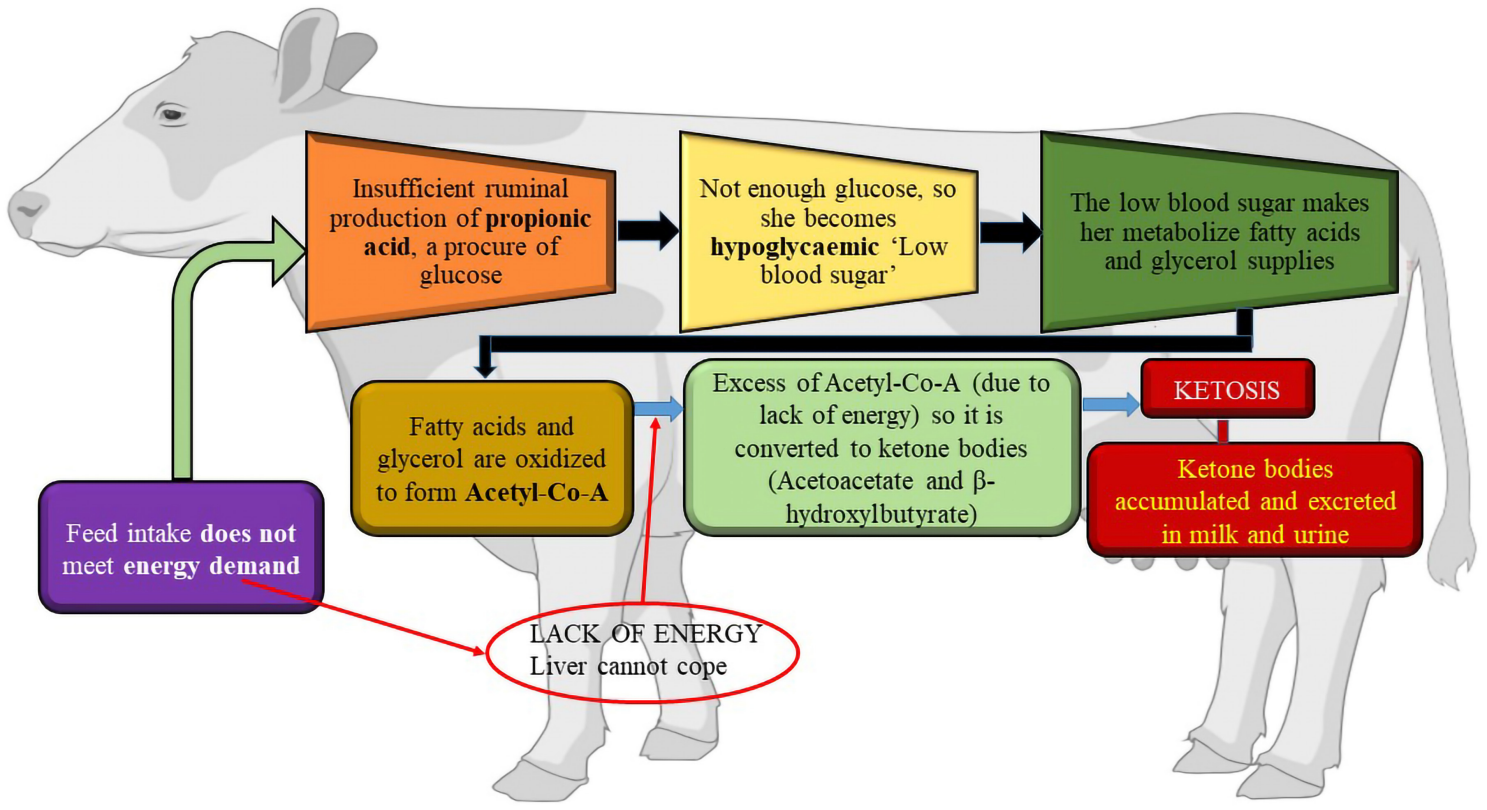
The etiology of Ketosis. Created with the web-based BioRender tool (BioRender.com).

During early lactation, the amount of energy required for the maintenance of body tissues and milk production exceeds the amount of energy obtained from dietary sources. Therefore, cows must utilize body fat as an energy source. There is a limit to the quantity of fatty acids that the liver tricarboxylic acid cycle can completely oxidize or export as very-low-density lipoproteins. When this threshold is crossed, acetyl-coenzyme A, which is not integrated into the tricarboxylic acid cycle, is transformed into acetoacetate and beta-hydroxybutyrate, and triglycerides build up inside hepatocytes, hindering their ability to operate. These ketone bodies are indicative of ketosis and may be observed in the blood, milk, and urine. They typically appear clinically between 10 days and 3 weeks after calving. Hypoglycemia is the outcome of poor gluconeogenesis. Cows experience increased depression, which lowers milk output and feed intake. The capacity of an over conditioned cow's liver to oxidize fatty acids is more constrained than that of a leaner animal (49). When the body's natural defenses against a negative energy balance are compromised, cows develop ketosis (50). While inadequate feedback control of non-esterified fatty acid release from adipose tissue is a likely contributing factor to ketosis, failure of hepatic gluconeogenesis to deliver sufficient glucose for lactation and body demands may be a cause of ketosis (50). After calving, all dairy cows typically undergo a phase of negative energy balance and fat mobilization; however, not all animals develop hyperketonemia, and even fewer develop clinical ketosis. Different animals respond differently to negative energy balance in their metabolism. Nevertheless, insufficient metabolic adaptability appears to be linked to the onset of ketosis, rather than the severity of low-energy status alone. Ketosis can develop during the early stages of lactation for various reasons. Early-lactating cows are more susceptible to ketosis if they have a high body condition score (greater fat storage) and significant fat mobilization around parturition (51–53). The likelihood of peripartum diseases causing cows to consume less feed raises the possibility of ketosis. For instance, there is a link between an increased risk of disease, lameness (54), and milk fever (55). Environmental variables contribute to the genesis of ketosis in addition to several “cow factors”. The increased demand for glucose during peak lactation or the initial stages of milk production in high-yielding animals restricts oxaloacetate availability for metabolic reactions. This leads to decreased tricarboxylic acid cycle activity, which in turn lowers the rate of acetyl-CoA catabolism and energy production. Increased breakdown of depot fats due to restricted energy production results in increased acetyl-CoA synthesis. Ketone body production is the target of extra acetyl-CoA. The blood ketone content increases, resulting in ketonemia, and ketone bodies are present in the urine, causing ketonuria due to the decreased ability of extra-hepatic tissues to catabolize acetyl CoA. The combined condition of ketonemia and ketonuria is known as ketosis. The composition and quality of animal feedstuff may play a role in the development of ketosis. Figure 3 shows the etiology of ketosis in cows.

### 3.2 Clinical presentation

Ketosis manifests in two distinct phases: the subclinical or latent phase, and the clinical phase characterized by apparent symptoms. Subclinical ketosis in dairy cows refers to the occurrence of ketone bodies in the bloodstream without any observable symptoms of ketosis (56). Subclinical ketosis, also known as the latent phase, is characterized by non-specific disruptions in metabolic balance. The symptoms of this disorder encompass imbalanced milk production, a propensity to experience weight loss despite a normal appetite and functioning rumen, as well as occasional cases of mild ketonuria. A cow exhibiting subclinical ketosis may not always display thinness, as shown in cases of fat cow syndrome. Milk production imbalance is defined as substantial daily fluctuations in milk amount without any discernible explanation (57). Subclinical ketosis is characterized by low blood sugar levels (hypoglycemia) and the presence of ketones in the blood (ketonemia) from a biochemical perspective (58). Subclinical ketosis remains latent until the level of ketones in the blood exceeds the level of glucose, and the level of urinary protein also changes (59). Subclinical ketosis is of great practical and economic relevance because of its hidden progression and large impact on production losses. The occurrence of subclinical ketosis in dairy cows during the early lactation period varies between 7.5% and 14%. Cows experiencing subclinical ketosis have a 4.9-fold increased likelihood of having metritis, a 6.1-fold increased likelihood of developing displaced abomasum, and a 1.98-fold increased likelihood of developing hoof diseases (60). The clinically manifested phase occurs when the level of ketones in the blood exceeds the level of glucose, however it may potentially occur sooner if hormonal control is ineffective. Initially, the clinical presentation is mainly characterized by non-specific symptoms of impaired energy balance and indigestion syndrome. As a result, early lactation leads to rapid weight loss, lower milk output, reduced fertility, increased veterinary expenses, and eventually a higher percentage of cows being culled. Body temperature can vary, ranging from normal to lowered, mostly as a result of diminished oxidative processes in the body. Bradycardia and bradypnea can manifest (61). The majority of animals display lethargy, experiences a decrease or fluctuation in appetite, and may demonstrate aberrant behavior. The normal functioning of the rumen is disrupted, resulting in either acidity or blockage. Animals have the ability to release a scent of acetone, particularly in their breath and milk, which also has a taste that is bitter. In instances of extreme severity, the entire herd may have an acetone odor (62, 63). These symptoms arise due to an elevation in ketone levels in the bloodstream, rumen contents, and urine. The content of ketones in the blood can increase by 5 to 10 times compared to the normal level, while in urine, it can rise by 10 to 100 times (56). If ketone bodies are detected in the urine on the 21^st^ day of the disease, and appropriate preventative measures and treatment protocols have been implemented prior to that, it is recommended to euthanize such a cow (64). In situations that are more severe and progressed, ketosis may be accompanied by symptoms of metabolic acidosis, dehydration, and central nervous system abnormalities. Acidosis occurs due to the buildup of acidic metabolic by-products and a decrease in alkaline reserves. Depletion of sodium ions results in loss of water from the body (45). Dehydration is characterized by an increase in the concentration of blood components and a relative increase in protein levels. The skin's elasticity is diminished, and the eyes appear to be sunken. Central nervous system symptoms occur as a result of decreased oxidative processes in the nervous system caused by a lack of carbohydrates, as well as the harmful effects of metabolic by-products, particularly acetoacetic acid. In acute cases, distinct signs of nervous system disorders manifest, including agitation, paralysis, abnormal sensations, and hyperesthesia. Animals engage in behaviors such as chewing without any food, grinding their teeth, making loud sounds, displaying a crazy expression, and having saliva dripping from their lips. Post-partum ketosis, a condition comparable to puerperal paresis, can develop after calving. The symptoms are identical to puerperal paresis, except that the pupillary reflex remains intact, and treatment with calcium is not beneficial (65). Animals in advanced stages experience a state of stupor, which eventually progresses into a coma. The prognosis and outcome of the illness are significantly influenced by symptoms emanating from the liver. According to Djoković et al. (66), moderate episodes of ketosis often do not exhibit clinical or biochemical indications of liver damage. Severe and advanced cases of liver disease, particularly if it is a long-term condition, may exhibit an enlarged liver that is somewhat sensitive to touch and may be detected by percussion and palpation. This is typically noticed in the area beyond the right final rib (67). From a histological perspective, the liver exhibits fatty degeneration, also known as steatosis. In more severe instances, cirrhosis may develop.

### 3.3 Clinical findings

Subclinical cases of ketotic diseases may alone be identified through systematic tests of urine and milk to detect the presence of ketone bodies. Animals in a herd or flock are chosen for testing based on a clinical assessment and statistics on milk production. An imbalance in milking over the whole lactation period often suggests a disruption in metabolic equilibrium (68). The gold standard for diagnosing subclinical ketosis in the laboratory is by measuring the concentration of BHBA in the blood and doing a liver biopsy (69). Subclinical ketosis is diagnosed when the blood concentration of BHBA exceeds 0.85 mmol/L. Studies have demonstrated that around 15% of herds exhibit ketosis, with a threshold of 10% considered to be important (70). The blood content of BHBA rises after eating, and samples should be collected at intervals of 4 to 5 h following the initial meal. The sample should comprise 12 randomly selected cows from a population of 50 cows. If one or two samples provide positive results, showing high levels of BHBA, it means that the herd's condition is in a critical state. If two or more cows in a group of twelve test positive, the herd sample is classified as positive for ketosis (43). The blood (and maybe milk and urine) can be analyzed to assess the levels of acetoacetic acid (71). Subclinical ketosis is defined as having an acetoacetic acid concentration over 0.36 mmol/L, whereas clinically exhibited ketosis occurs when the concentration of acetoacetic acid surpasses 0.5 mmol/L (72). Acetoacetic acid is an unsuitable laboratory measure for detecting ketosis due to its inherent instability, leading to its fast decomposition into acetone and carbon dioxide (73). The ketone bodies in milk have a concentration that is around 50% less than the concentration in blood. On the contrary, the concentration of ketone bodies in urine is many times larger than that in blood (74). An elevation in the levels of ketone bodies in the bloodstream corresponds with a reduction in the concentration of blood glucose. The typical blood glucose concentration in cattle varies from 2.3 to 4.1 mmol/L. If the concentration falls below 2.3 mmol/L, the animal is diagnosed as ketosis (75). Another significant indicator is NEFA. The NEFAs threshold value in cows 2 to 14 days before to calving and 4 to 5 h after the first meal of the day is >0.4 mEq/L (76). Proper storage of samples is essential, requiring them to be kept at a low temperature or even frozen from the time of collection until they are delivered to the laboratory. The presence of ketone bodies in urine may be identified by utilizing sodium nitroprusside, which is the approved test according to Kumar (77). The presence of ketones in urine is detected by observing a color shift, which can range from pink (+) to pink/purple (++) or dark purple (+++), depending on the concentration of ketones. Currently, there are several efficient techniques available for promptly identifying ketones in milk and urine, often referred to as dipstick tests. These tests enable the direct detection of acetone and acetoacetic acid in milk and urine (69).

### 3.4 Treatment and prevention

Ketosis in cows is a prevalent metabolic disorder that demands well-thought-out treatment strategies. The most common and effective treatment is oral administration of 250–400 g propylene glycol (drenched) every 24 h for 3 to 5 days. Extra treatment is advised in instances of hypoglycemia, such as vitamin B12 (1.25 mg, IM, every 24 h for 3 days) and bolus glucose treatment (500 mL of 50% dextrose solution, IV, as a single bolus) in neurologic patients. Restoring normoglycemia and lowering blood ketone body concentration are the goals of ketosis treatment. Another popular treatment is bolus glucose, which involves intravenous injection of 500 mL of 50% dextrose solution (78, 79). It is important to ensure that this solution is administered IV because it is highly hyperosmotic and causes severe tissue swelling and irritation if administered perivascularly. When type I ketosis (around peak lactation) occurs, bolus glucose therapy usually produces a quick, transient recovery. However, relapses are frequent and the benefits are typically fleeting. While dextrose administration is advised in cases of nervous ketosis, it may not be required or even beneficial in all cases of ketosis. As there is minimal evidence of benefits and some indications of risk, the administration of glucocorticoids is not advised. Propylene glycol functions as a precursor for glucose and treating ketosis with oral drenching (250–400 g [8–14 oz], PO, every 24 h for 3–5 days) is beneficial. Propylene glycol overdosing leads to CNS depression. In addition, oral propylene glycol drenching is supported when used in conjunction with vitamin B12 (1.25 mg, IM) for 3 days, especially in ketotic and hypoglycemic cows. Type II ketosis, which develops in the first 2 weeks post-partum, is often more resistant to treatment than type I ketosis, which develops closer to the peak of lactation. Frequently, a recurrent 5-day oral propylene glycol immersion regimen, frequently in conjunction with vitamin B12, appears to address these persistent ketosis issues (80). Although there is not much evidence to support this hypothesis, some studies have proposed that, in certain situations, a long-acting insulin preparation (150–200 U, IM, every 24 h for 5 days) would be helpful. Insulin should be administered in conjunction with glucose or a glucocorticoid to prevent hypoglycemia since it inhibits both adipose mobilization and ketogenesis. It is not authorized to use insulin in this manner.

## 4 Milk fever

Reduced blood calcium levels (hypocalcemia) are the hallmarks of milk fever, post-parturient hypocalcemia, or parturient paresis, an illness that mostly affects dairy cattle (81), but can also affect beef cattle and other domesticated animals that are not cows. It is also referred to a condition of low blood calcium levels without a fever, commonly known as milk fever, typically connected to parturition and the start of breastfeeding. It occurs at the beginning of lactation, after parturition, when the body's need for calcium to produce milk and colostrum surpasses its capacity to do so. “Fever” is a misnomer since the illness typically does not cause an increase in body temperature (82). Due to their lowered capacity to release calcium from the bone, older animals and specific breeds such as Channel Island breeds, are more likely to have milk fever (83).

Several factors affect the ability of cows to regulate the blood calcium process effectively, including age, because older cows are less able to mobilize calcium from their skeletons. A high level of estrogen around calving, coupled with less feed intake, inhibits calcium mobilization. Bone resorption of calcium is inhibited in cows fed high-potassium or high-sodium diets, owing to metabolic alkalosis (82). Sansom et al. (84) also reported that low magnesium intake reduced the ability of the gut to absorb calcium. Additionally, problems associated with digestion, such as acidosis and profuse diarrhea, reduce the amount of calcium available for absorption (29). When dry cows are fed grass instead of preserved feed, hypocalcemia appears to occur more frequently, especially during extended wet weather periods. Breed is another risk factor; the Jersey breed and, to a lesser extent, the Guernsey breed are especially prone to milk fever. This is most likely caused by the comparatively high production levels of these small breeds. Along with the number of lactations, the incidence of the disease increases. The increased need for calcium after calving due to increased milk output and the declining capacity of the aging process to release calcium from the skeleton are theorized to be the causes. Although hypophosphatemia and hypermagnesemia are frequently associated with hypocalcemia, hypomagnesemia can also result from inadequate magnesium intake, which can cause milk fever.

Preventing milk fever is crucial for maintaining the health, productivity, and profitability of dairy herds, particularly in high-producing cows. Milk fever occurs when blood calcium levels drop sharply around calving, leading to muscle weakness, reduced feed intake, and impaired milk production. Severe cases can result in recumbency, secondary metabolic disorders (e.g., ketosis, mastitis), and even death. Preventive measures, such as optimizing the dietary cation-anion difference (DCAD) during the prepartum period, have proven highly effective. By feeding anionic salts (e.g., calcium chloride) to induce mild metabolic acidosis, calcium mobilization from bones and intestinal absorption are enhanced, reducing the risk of hypocalcemia (85). Additionally, oral calcium supplements administered at calving and 12 h post-partum can provide immediate calcium support, particularly for high-risk cows. Studies have shown that these strategies not only prevent clinical milk fever but also reduce the incidence of subclinical hypocalcemia, which is associated with decreased milk yield and reproductive performance (86, 87). By prioritizing milk fever prevention, dairy producers can improve cow health, enhance milk production, and ensure sustainable herd management.

### 4.1 Etiology

Hypocalcemia or insufficient calcium in the blood serum is the cause of milk fever. At the end of the dry season, the metabolism of dairy cows quickly shifts from a resting phase to a high-performance phase. During the dry season, cows do not require as much Ca.

**Figure 4:**
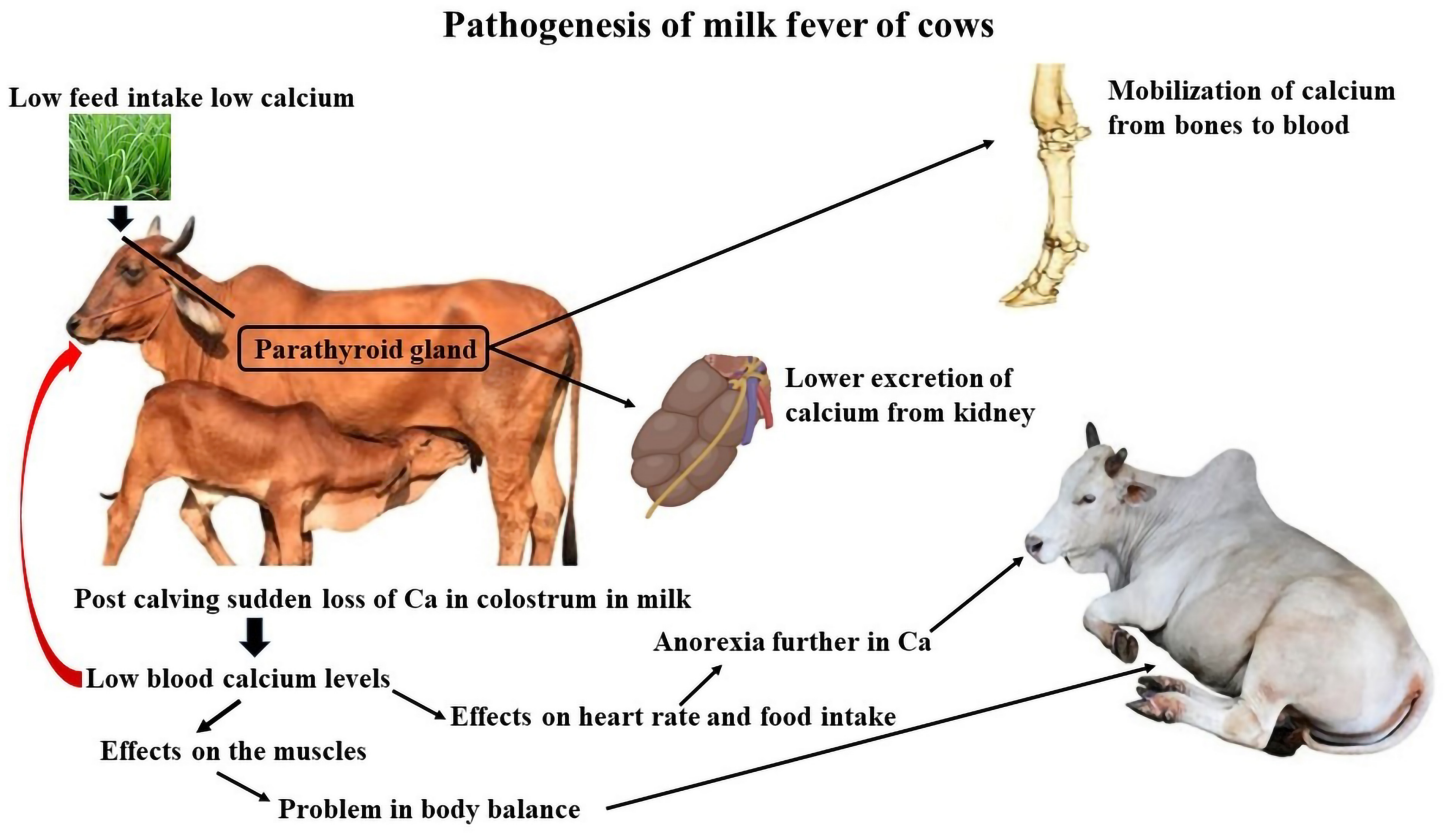
Pathogenesis of milk fever of cows. Created with the web-based BioRender tool (BioRender.com).

The need for calcium nearly doubles when lactation begins because colostrum synthesis requires a large amount of calcium (2.3 g/L). Typically, bones or food supplies calcium. Mobilization frequently fails to be initiated rapidly enough in elderly cows. Because there is not enough calcium from the feed and bones, the body takes what is needed from the muscles. This causes overstimulation of the nervous system and paralysis (Figure 4). Hypocalcemia first results in hyperexcitability of the neurological system, which usually leads to paresis and weaker contractions of the muscles (23). Cows of any age can develop parturient paresis, although high-producing dairy cows that start their third or later lactation are more likely to experience parturient paresis. Breeds, such as Guernsey and Jersey, have higher incidence rates.

### 4.2 Clinical presentation

Recumbency around the time of parturition and hyperexcitability that leads to flaccid paralysis are possible signs. The majority of parturient paresis occurrences occur within the first 3 days following parturition. Hypocalcemia not only causes parturient paresis but can also lead to metritis, abomasal displacement, retained fetal membranes, dystocia, and mastitis. There are three distinct phases of parturient paresis.

Stage 1 cows exhibit clinical signs of hypersensitivity and excitability, although they remain mobile and upright. They may show signs of head swaying and ear twitching, as well as minor ataxia and tiny tremors over the flanks and triceps. Cows may snort, look agitated, or shuffle their feet. Cows will probably move on to a more severe stage 2 if calcium treatment is not initiated during stage 1.

Cows with stage 2 parturient paresis are paretic to the point where they cannot stand. Their musculoskeletal control is adequate to sustain sternal recumbency. They usually have cold extremities, dry snouts, subnormal body temperatures, and obtundation and they are also anorectic. Owing to compromised cardiac contractility, auscultation demonstrates tachycardia and a decrease in the loudness of the heart sounds. Weak peripheral pulse may be observed. Gastrointestinal stasis, which can cause bloating, inability to pass gas, and loss of anal sphincter tone, is caused by smooth muscle paralysis (88). Rectal examination may reveal a dilated bladder as a sign of an inability to urinate. Asymmetry of the cervical musculature may result in an S-shaped curvature of the neck may develop, indicating muscle weakness, if the cow can extend its head.

Cows with stage 3 parturient paresis progressively lose consciousness to the point of coma. They cannot lie flat on their sides or sustain sternal recumbency (85). They can have considerable bloats and extremely flaccid muscles, and may not respond to stimulation. Peripheral pulses may become undetectable and the heart rate may approach 120 bpm as the cardiac output deteriorates. Cows in stage 3 may not survive for more than a few hours without treatment.

### 4.3 Clinical diagnosis

Prior to therapy, it is imperative to get a blood sample in order to validate the diagnosis by the identification of a reduced calcium level in the serum. Cows with blood calcium levels below 8.0 mg/dL are classified as hypocalcemia. For optimal results, it is recommended to collect the blood sample from the cow 12 to 24 h after giving birth. It is also important to collect at least 12 samples in order to obtain reliable data (89). If three to five samples have a serum calcium value below 8.0 mg/dL, they are deemed to have reached a critical threshold. If six or more cows out of a group of twelve test positive, the sample is considered positive, indicating that the herd is experiencing hypocalcemia (90). Urine pH has demonstrated efficacy as a diagnostic technique for dietary cation-anion balancing DCAD verification, as blood pH is expected to be about 7.0. For diagnostic reasons, a minimum of eight urine samples from cows is required, and it is recommended to do testing on a weekly basis, or even more regularly. The process is simple, as it just requires the use of a standard pH indicator paper (91).

### 4.4 Treatment

The treatment involves administering calcium borogluconate intravenously at a dosage of 1 gram of calcium per 45 kilograms of the animal's body weight. For the management of big cows that produce a significant amount of milk, an extra dose of the medication might be given by injecting it under the skin (92). Due to the cardiotoxic nature of calcium, it is important to deliver the medicine at a very slow rate, taking 10 to 20 min, while closely monitoring the heart's activity using auscultation (93). If bradycardia or arrhythmia is observed, cessation of therapy is necessary, and resumption should only occur gradually until the heart's activity returns to normal. Administering calcium intravenously is not advisable for the treatment of hypocalcemic cows that are still able to stand. Administering calcium intravenously promptly elevates blood calcium levels, posing a possible risk (93). Elevated concentrations of calcium in the bloodstream can lead to life-threatening cardiac problems and inhibit the movement of calcium in cows during crucial periods (94). Due to compromised peripheral absorption, the subcutaneously injected drug is not recommended as the sole option. Administering calcium subcutaneously restores blood calcium levels to normal after 6 h of injection (92). According to Miltenburg et al. (95), giving cows with low calcium levels oral calcium supplements is the most effective option, especially if the cows are still able to stand. According to Oetzel (96), cows that show a positive response to intravenous calcium therapy and are able to regain consciousness and able to swallow should also be provided with oral calcium supplements 12 h following the treatment. Table 2 provides a comprehensive comparison.

**Table 2:** Comparison of calcium supplementation strategies for milk fever prevention.

**Strategy**	**Method**	**Efficacy**	**Advantages**	**Limitations**	**Best practices**	**References**
Oral calcium boluses	Administer calcium chloride or calcium propionate at calving and 12 hours post-partum.	Reduces clinical milk fever by 50% and subclinical hypocalcemia by 30%.	Immediate calcium support; easy to administer.	Risk of hypercalcemia with over-supplementation.	Use for high-risk cows; combine with DCAD diets for optimal results.	(85, 128)
DCAD diets	Prepartum diets with negative DCAD (100.0 to −150.0 mEq/kg DM) using anionic salts.	Reduces clinical milk fever by 70% and subclinical hypocalcemia by 50%.	Enhances calcium mobilization; long-term prevention.	Requires careful monitoring of urine pH; risk of over-acidification.	Maintain urine pH between 6.0–6.5; combine with oral calcium for high-producing herds.	(85, 129)
Intravenous calcium	Administer calcium gluconate intravenously for clinical cases.	Rapidly restores blood calcium levels in clinical cases.	Immediate effect in emergencies.	Does not prevent milk fever; risk of cardiac arrhythmias if improperly dosed.	Reserve for clinical cases; supplement with oral or dietary strategies for long-term prevention.	(130)

### 4.5 Prevention

Implementing measures to prevent milk fever is financially advantageous for dairy farmers as it leads to decreased losses in production, mortality, and veterinary costs related to milk fever. Various nutritional strategies have been employed to regulate hypocalcemia and facilitate calcium release from dairy cattle. These strategies include administration of anionic salts, diets low in calcium ions, and supplementation with vitamin D (97, 98). Prior to conception, consuming diets deficient in calcium enhances the secretion of parathyroid hormone. This process activates osteoclasts within the bone, enhances the absorption of calcium within the bone, encourages the renal tubules to reabsorb calcium from urine, and initiates the production of 1,25-dihydroxyvitamin D. Consequently, after the onset of lactation, calcium regulatory pathways become activated and can avoid low levels of calcium in the blood (88). According to Goff and Koszewski (99), one technique for reducing milk fever in cows is to provide diets that are low in calcium throughout the dry period. This can be accomplished by consuming < 50 grams each day. Consequently, forage that is high in calcium, such as alfalfa, should be eliminated from the animal's diet. Regular feeding of corn silage and grass hay throughout the dry months is recommended to reduce calcium levels effectively (100). Bhanugopan and Lievaart (101) discovered that all farmers employed hay, straw, and grain as a comprehensive nutritional regimen throughout the dry period. Grain feeding facilitates rapid adaptation of the rumen to high-energy meals provided after post-partum, and grains also possess low calcium content.

#### 4.5.1 Dietary cation-anion balancing

A dietary approach aimed at preventing milk fever during the early stages of lactation while also improving the overall health and performance of cows (102). One often-used preventive technique is the administration of anionic salts to reduce the difference between cations and anions in the diet. This strategy has been effectively used in dairy farms (103). The aim of this type of supplementation is to reduce the intake of cations that can be absorbed, such as sodium and potassium, while increasing the intake of anions that are easily accessible, such as chlorine and sulfur monoxide (85). To prevent milk fever, it is advisable to limit the amount of dry fodder administered to cows because of its high potassium content. It is essential to include silage and succulent/green fodder in a significant portion of the dry cow's diet, as they have lower potassium levels (104). Bhanugopan and Lievaart (101) proposed that a way to avoid milk fever in cows is to give them calcium supplements orally before and after parturition. A study conducted by Amanlou et al. (92) found that administering two subcutaneous calcium infusions within the first 18 h after giving birth can reduce the likelihood of post-partum disorders (such as metritis, clinical and subclinical endometritis, and hypocalcemia) in cows. The experimental group, which received infusions, showed a lower risk than the control group.

Magnesium plays a crucial role in calcium metabolism, particularly in the process of calcium resorption from the bone through the action of parathyroid hormone. In a study published in 2018, Goff and Koszewski (99) emphasized the importance of magnesium supplementation in avoiding milk fever. A study conducted by DeGaris and Lean (83) concluded that increasing magnesium supplementation is the most effective approach for avoiding milk fever. Furthermore, Bhanugopan and Lievaart (101) suggested the use of vitamin D supplementation in pre-partum dry cows, in addition to previously described approaches. This approach requires the administration of up to 10 million international units (IU) of vitamin D each day for a period of 10–14 days before giving birth, thereby enhancing the absorption of calcium in the intestines. Østergaard et al. (105) and Mulligan and Doherty (4) found that dairy cows that are over-conditioned during calving had a higher risk of developing milk fever. This is because they have a greater amount of calcium released in their milk, up to four times more than cows with a normal body condition score (BCS). Compared to leaner dairy cattle, excessively obese cows experience reduced food consumption during the final week or 10 days prior to giving birth. This might lead to a decrease in calcium and magnesium consumption to levels that make them more susceptible to hypocalcemia. Therefore, it is crucial to prevent the dry cows from becoming excessively overweight. Cows that have had substantial body condition deterioration during the dry period are also prone to developing milk fever (106).

## 5 Hypomagnesemia

Hypomagnesemia is an electrolyte imbalance characterized by low blood magnesium levels. When the amount of magnesium lost by sweat, urine, milk, or digestive secretions exceeds the amount of magnesium consumed through nutrition, hypomagnesemia results. In contrast to calcium or phosphorus, magnesium cannot be reabsorbed from the bone by dairy cattle to supplement magnesium during transient magnesium insufficiency. Animals may experience severe muscle weakness, convulsions, or spasms in acute clinical situations. They may also pass away. It is uncommon for lactating dairy cows to experience acute hypomagnesemia; however, hypocalcemia, or milk fever are much more common reactions. Hypomagnesemia is a common occurrence during calving, which is often confused with milk fever.

### 5.1 Etiology

When the body's absorption of dietary magnesium is insufficient to meet the needs for breastfeeding (120 mg/kg milk) and maintenance (3 mg/kg body weight), hypomagnesemic tetany results from a decrease in the plasma magnesium concentration. This might happen when cows graze short grass-dominant pastures with < 0.2% magnesium on a dry matter basis, during transit, or when they reduce their food intake due to bad weather. During breastfeeding, live weight loss occurs when there is insufficient herbage availability (< 1,000 kg dry matter/hectare). The body mobilizes its magnesium stores to support lactation more than it actually needs to, leading to reduced plasma magnesium levels (107).

**Figure 5:**
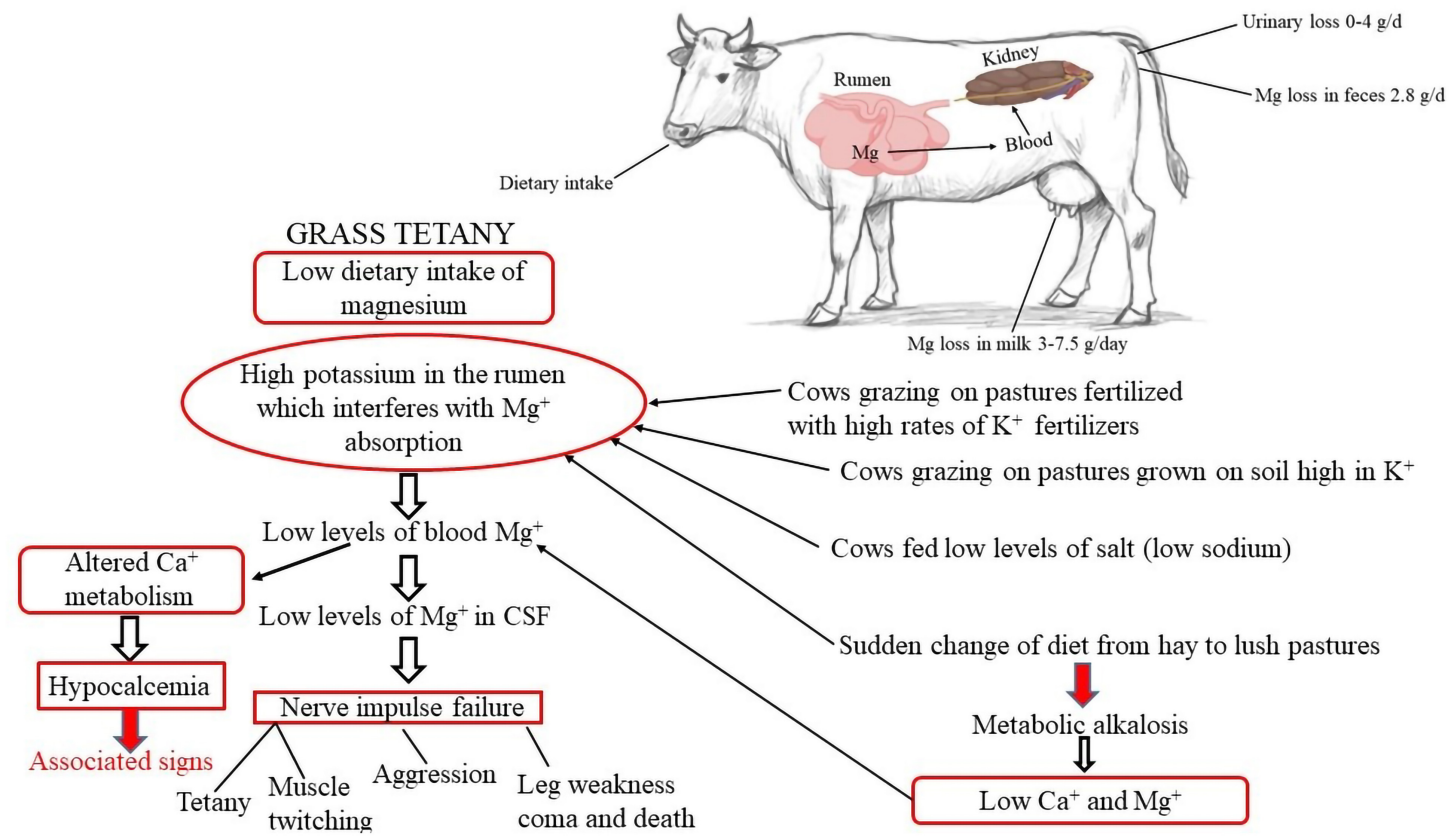
Etiology of hypomagnesemia. Created with the web-based BioRender tool (BioRender.com).

Secondary hypomagnesemia stems from dietary variables that hinder Mg absorption, such as excessive K and N concentrations or low Na levels (103, 108). Fertilizers containing nutrients such as nitrogen (N), potassium (K), and phosphorus (P) can enhance forage production. However, they may reduce the bioavailability of magnesium (Mg) in the rumen, as illustrated in Figure 5 (103, 108). High N content in fast-growing grasses (ranging from 4.2% to 6.3%, equivalent to 26%−39% crude protein) leads to high ammonium ion concentrations in the rumen, increasing pH and decreasing Mg bioavailability (109). Until blood calcium concentrations are also lowered to < 0.8 mg/dL (0.35 mmol/L), which frequently happens in cattle grazing green cereal crops, cows frequently do not exhibit clinical symptoms of hypomagnesemic tetany. A decrease in calcium intake, calcium absorption, or both can lead to hypocalcemia. Cattle raised in lush grass pastures or green cereal crops are more likely to have metabolic alkalosis (urine pH > 8.5), which can lead to a reduced supply of ionized calcium and magnesium, thereby raising the risk of hypocalcemia and hypomagnesemia. In cows with hypomagnesemia, urine magnesium concentrations are undetectable and yet serve as good indicators of magnesium status (107).

### 5.2 Clinical presentation

Hypomagnesemia in cows' manifests in several distinct ways. Neurologically, lactating cows, especially those grazing on lush grass pastures or green cereal crops, are at risk (107, 110). They often exhibit hyperexcitability, with sudden head-throwing, loud bellowing, and erratic galloping. Muscular spasms are common, and in severe cases, seizures occur. During seizures, cows may display paddling motions, chomping jaws, frothy salivation, and eyelid fluttering. In extreme situations, respiratory distress sets in, potentially leading to collapse and death. Digestively, affected cows show a significant reduction in appetite, either refusing to eat or consuming only small amounts of feed. This can lead to weight loss and malnutrition. Diarrhea, with thin or watery feces, is also a prevalent digestive symptom (111). Reproductively, the deficiency of magnesium ions impacts the cows' breeding capacity, disrupting normal physiological processes and causing a decline in their overall reproductive function (107).

### 5.3 Clinical diagnosis

Clinical symptoms emerge abruptly in acute cases. Affected animals exhibit unease, stop eating and remain wary. These symptoms are associated with muscular twitching. The animal may seem terrified or “agitated”. Faltering gait can lead to falls and spastic limb movements in the unwell animal. Clinical symptoms can cause death within 4.0 h. A rapid increase in body temperature may occur after a tetanic event. The pulse and respiration increase. In sub-acute situations, progressive appetite loss leads to unpredictable conditions. Research indicates a 22–55 kg body weight reduction in pregnant animals and a 27% decline in milk supply in nursing animals.

Clinical signs of hypomagnesemic tetany in sheep occur when hypomagnesemia (plasma total magnesium [tMg] < 0.5 mg/dL [0.2 mmol/L]) occurs concomitantly with hypocalcemia (plasma total calcium [tCa] < 8.0 mg/dL [2.0 mmol/L]). The disease in lactating ewes occurs under essentially the same conditions and has the same clinical signs as those of cattle. Treatment response is typically used to confirm the diagnosis, followed by confirmation of pre-treatment sample hypomagnesemia. The reference range for total magnesium in sheep is 2.2–2.8 mg/dL (0.9–1.15 mmol/L) and in cattle is 1.8–2.4 mg/dL (0.75–1.0 mmol/L). In sheep and cattle, tetany typically develops when plasma tMg is < 0.5 mg/dL (0.2 mmol/L) and < 1.2 mg/dL (0.5 mmol/L). Hypocalcemia frequently occurs concurrently with other conditions. In cows with hypomagnesemic tetany, magnesium is typically not detected in the urine (107). Mg concentrations in dead animals may be normal due to muscle damage and leakage from intracellular pools. Mg concentrations < 1 mg/dL (0.4 mmol/L) from the CSF within 12 h of death or from the vitreous humor of the eye within 24–48 h after death are indicative of hypomagnesemic tetany, provided environmental temperatures have remained below 23°C (107).

### 5.4 Treatment

Clinical cases are treated with intravenous, subcutaneous, or oral Magnesium solutions. Recommended intravenous Mg dosage: 2.0–4.0 g. Alternatively, 200.0–400.0 mL of 25% Magnesium sulfate (MgSO_4_7H_2_O) can be subcutaneously injected at 100 mL each location. The animals in coma fail treatment. Oral Mg salts can maintain plasma Mg levels for longer and lower the incidence of aspiration pneumonia once the animal has recovered esophageal reflexes (112). Oral administration of Mg is not recommended as an initial therapy due to its slow absorption. Intraruminal gavage with an aqueous solution comprising 100.0 g MgO, 100 g CaCl_2_, and 50 g NaCl may be more effective in hypocalcemia with hypomagnesemia. Na boosts ruminal Magnesium absorption. Because it is better absorbed than MgO, 200 ml of 50% MgSO_4_7H_2_O intraruminal restores blood Mg levels faster (112). When an animal exhibits clinical symptoms, it must be treated immediately with calcium and magnesium solutions, ideally administered slowly intravenously while the heart is being watched. Because it takes longer to recover magnesium levels in the cerebrospinal fluid (CSF), animals with hypomagnesemic tetany respond to treatment more slowly than animals with hypocalcemia alone. Stimulation of the animal while receiving treatment can produce lethal seizures. A mature cow needs 1.5–2.25 g of elemental magnesium (107). This equates to 15–22.5 g of Mg sulfate solution or 30.0–45.0 ml of a 50% Mg sulfate solution because Mg sulfate is only 9.7% elemental Mg. Mg is present in several commercial intravenous formulations as a solution of hypophosphite, borogluconate, and chloride. A standard course of treatment involves slow intravenous injection of 400.0 ml of 40% calcium borogluconate and 50.0 ml of 25% magnesium sulfate. If possible, cows should be relocated off the tetany-prone pasture after treatment and given time to respond naturally. The illness can return within 36.0 h following the first treatment if the animals are not given hay treated with 60.0 g of magnesium oxide every day.

### 5.5 Prevention

Prevention of hypomagnesemia in cows mainly lies in the rational adjustment of the diet structure. During the periods prone to the disease, such as winter or when cows are pregnant and lactating, it is essential to ensure an adequate supply of magnesium in the diet (110, 113). Research shows that starting to supplement high—magnesium mineral feed at least 30 days before calving is significantly effective. Cows need to consume 4.0 ounces of a mineral mixture containing 12% magnesium per day. For example, an appropriate amount of magnesium oxide (MgO) can be added to grains to ensure the magnesium intake of cows. However, magnesium oxide has a bitter taste and poor palatability. To improve the cows' feeding enthusiasm, it can be mixed with palatable ingredients such as molasses and concentrate (110, 113, 114).

In terms of pasture management, fertilization should be carried out reasonably according to the results of soil testing, and excessive application of potassium fertilizer should be avoided. Because the potassium content in lush spring forage is too high, which will hinder the absorption of magnesium by cows (115). In addition, during the periods when cows are prone to hypomagnesemia, some targeted measures can be taken. For example, providing hay for cows or limiting the grazing time to 2.0–3.0 h per day can slow down the passage of food through the digestive tract and promote the absorption of magnesium (116, 117). At the same time, for pastures with a high risk, animals that are less likely to be affected (such as heifers, dry cows, and fattening cattle) should be given priority for grazing. Some studies point out that 60.0 grams of magnesium oxide can be supplemented to cows daily for prevention during the dangerous period. The magnesium content in forage can also be increased by spreading magnesium oxide powder (500 grams per cow) on hay or spraying a 2% magnesium sulfate solution every 1.0–2.0 weeks. However, it should be noted that if the rainfall exceeds 40.0–50.0 mm within 2.0–3.0 days after spraying, the treatment needs to be carried out again.

## 6 Improvement strategies

### 6.1 Nutritional optimization

#### 6.1.1 Balanced diet design

Formulating a diet that precisely meets the nutritional needs of cattle at different life stages is crucial (118). This involves carefully adjusting the ratio of forage to concentrate. During the transition period around calving, cows experience a sharp increase in energy requirements for milk production. Thus, providing a diet with an appropriate balance of readily available energy sources, such as high-quality grains and fats, while ensuring sufficient effective fiber to maintain rumen health is essential. For example, a diet rich in alfalfa hay, which provides both energy and fiber, can be beneficial. Additionally, ensuring an adequate supply of rumen-protected amino acids supports protein synthesis and overall metabolic function.

To prevent metabolic disorders, it's important to avoid sudden dietary changes. Instead, gradually introduce new feeds to allow the rumen microbiota to adapt. This helps maintain a stable digestive environment and reduces the risk of issues like acidosis, which can contribute to a cascade of metabolic problems (119).

#### 6.1.2 Mineral and vitamin supplementation

Cattle require a diverse range of minerals and vitamins for optimal health. Deficiencies in key minerals such as calcium, phosphorus, magnesium, and trace elements like selenium and zinc can significantly impact metabolic processes. For instance, calcium is vital during the periparturient period to prevent conditions related to low blood calcium levels. Supplementing with vitamin D can enhance calcium absorption, further supporting metabolic balance (120, 121).

In addition to major minerals, B vitamins play a crucial role in energy metabolism (122, 123). For example, vitamin B12 is involved in the conversion of propionate to glucose, which helps prevent the accumulation of ketone bodies and the development of ketosis-like conditions (124). Regular monitoring of the cattle's diet and blood mineral and vitamin levels allows for targeted supplementation as needed.

#### 6.1.3 Controlled feeding regimens

Implementing a consistent and controlled feeding schedule is essential. This includes monitoring feed intake closely and adjusting the amount of feed based on the cattle's body condition score and production stage (125). Feeding small, frequent meals helps maintain a stable rumen pH, promoting efficient digestion and nutrient absorption.

Moreover, proper management of feed quality is paramount. Ensuring that forage is fresh, free from mold and toxins, and stored correctly can prevent digestive disturbances that may lead to metabolic disorders. Additionally, providing clean and fresh water at all times is crucial for maintaining normal metabolic functions.

### 6.2 Transition period management

#### 6.2.1 Pre-partum and post-partum care

The transition period, spanning from a few weeks before to a few weeks after calving, is a critical time for cows. During this phase, providing a clean, comfortable, and well-ventilated housing environment is essential. This helps reduce stress, which can otherwise disrupt normal metabolic processes.

Pre-partum, gradually adapting cows to the post-partum diet minimizes rumen upset. Post-partum, close monitoring of the cow's health status, including body temperature, milk production, appetite, and behavior, is necessary. Early detection of subtle changes, such as a decrease in milk production or a change in appetite, can indicate the onset of a metabolic disorder and allow for prompt intervention.

#### 6.2.2 Hormonal regulation

Hormonal imbalances can contribute significantly to metabolic disorders in cattle. In some cases, hormonal therapies can be used to regulate the cow's physiological functions during the transition period. For example, medications that enhance insulin sensitivity can help improve glucose utilization and prevent the development of ketosis-like conditions. Additionally, hormones that regulate calcium metabolism can be administered to prevent issues related to low blood calcium levels.

However, the use of hormonal therapies should be carefully considered and guided by a veterinarian, as improper use can have adverse effects on the cow's health.

### 6.3 Health monitoring and disease prevention

#### 6.3.1 Regular veterinary examinations

Scheduled veterinary check-ups are vital for early detection of metabolic disorders. These examinations should include comprehensive blood tests to assess key metabolic parameters such as glucose, ketone bodies, liver enzymes, and mineral levels. Additionally, urine testing can provide valuable insights. Measuring the levels of specific proteins in urine, such as albumin and certain enzymes, can help detect kidney function abnormalities which may be associated with metabolic disorders (59). By regularly monitoring these parameters, subclinical metabolic disorders can be identified before they progress to more severe, clinical cases.

For example, detecting low magnesium levels in the blood through regular testing can allow for timely magnesium supplementation to prevent the development of hypomagnesemia, which can cause muscle.

#### 6.3.2 Vaccination and parasite control

Maintaining a robust vaccination program protects cattle from infectious diseases that can contribute to metabolic disorders. Diseases such as infectious bovine rhinotracheitis and bovine viral diarrhea can weaken the immune system, increase stress levels, and disrupt normal metabolic functions.

Effective parasite control, including regular deworming and tick control, is also crucial. Parasites can cause nutrient losses, anemia, and damage to the digestive tract, all of which can impact the cattle's overall health and metabolic processes.

### 6.4 Genetic selection and breeding

#### 6.4.1 Selection for metabolic resilience

Incorporating metabolic traits into the genetic selection process can lead to the development of cattle with enhanced metabolic resilience. Through genetic testing, cattle with favorable genetic markers related to insulin sensitivity, glucose metabolism, calcium homeostasis, and other metabolic functions can be identified and selected for breeding.

Over time, this approach can help reduce the prevalence of metabolic disorders within the herd. For example, selecting cattle with genetic traits that allow for more efficient calcium utilization can lower the risk of developing milk fever and other calcium-related metabolic issues.

#### 6.4.2 Breeding for adaptability

Choosing cattle breeds or lines that are well-adapted to the local environment and production conditions is another important strategy. Cattle that are better suited to the climate, available feed resources, and management practices are less likely to experience stress-induced metabolic disorders.

For instance, in regions with harsh winters, breeds with thick coats and higher cold tolerance may be more suitable, as they can maintain normal metabolic functions without expending excessive energy on thermoregulation.

### 6.5 Environmental management

#### 6.5.1 Temperature and humidity regulation

Extreme temperatures, whether hot or cold, can have a significant impact on cattle metabolism. In hot climates, providing shade, using sprinkler systems, and ensuring proper ventilation can help reduce heat stress (126). Heat stress can lead to decreased feed intake, altered metabolism, and an increased risk of metabolic disorders such as ketosis.

In cold climates, providing adequate shelter, warm bedding, and protection from the wind helps cattle conserve body heat. This reduces the energy required for thermoregulation and allows the cattle to maintain normal metabolic functions.

#### 6.5.2 Stress mitigation

Minimizing sources of stress in the cattle's environment is essential for maintaining metabolic health. Overcrowding, sudden changes in diet or management practices, and transportation can all cause stress, which can disrupt hormonal balance and immune function.

Implementing stress-reducing measures such as providing sufficient space, maintaining a consistent feeding and management routine, and using gentle handling techniques can help keep cattle calm and reduce the risk of metabolic disorders.

### 6.6 Personnel training and awareness

#### 6.6.1 Comprehensive training programs

Providing in-depth training to farm workers and managers on the prevention, detection, and treatment of metabolic disorders is crucial. Training should cover all aspects of cattle management, including nutritional principles, handling during the transition period, and interpretation of clinical signs.

Workers should be trained to recognize the early warning signs of metabolic disorders, such as changes in behavior, appetite, or milk production. They should also be knowledgeable about proper feeding practices, including how to adjust diets based on the cattle's needs and how to administer supplements correctly.

#### 6.6.2 Awareness campaigns

Conducting awareness campaigns within the farming community can help raise the overall level of understanding about metabolic disorders in cattle. These campaigns can use various channels, such as workshops, online resources, and printed materials, to disseminate information on best practices in cattle management, the economic impact of metabolic disorders, and the importance of early intervention.

By empowering farmers and farm workers with knowledge and skills, it becomes possible to implement effective prevention and management strategies, ultimately improving the health and productivity of the cattle herd.

## 7 Conclusion

Metabolic disorders are diseases related to the disturbance of one or more metabolic processes in an organism. The transition period, which includes 3 weeks before and 3 weeks after parturition, is critical for dairy cows. This period is associated with multiple changes, including hormonal changes, moving from a non-lactating to lactating state to a lactating state and a major drop in feed intake. A large proportion of the animal population is typically at risk of developing metabolic illnesses, which have a significant economic impact. Since the days leading up to calving and the early stages of lactation are crucial for female nutrition, food consumption cannot be affected during this time. Any condition that limits food intake during this phase (such as ketosis or milk fever) accelerates the body's metabolism when the animal needs energy, which causes fat to accumulate in the liver and impairs the ability of females who are already low in body mass. The profitability of dairy farms depends on effective reproduction, which is correlated with both nutritional status and metabolic health. Periparturient cows have reduced pregnancy rates per insemination, delayed return to ovulation, and a higher rate of pregnancy loss. Consequently, it is anticipated that implementing dietary and wellness initiatives that lower the likelihood of metabolic disorders will improve cow health and increase fertility. During the transition period, high energy demand combined with low food intake increases the likelihood of developing metabolic problems. Dietary formulations to reduce the degree and extent of negative nutrient balance, enhance Ca homeostasis, and lessen the severity of negative energy balance are strategies to control peripartum metabolic health.
